# Setting Up an Undergraduate Immunology Lab: Resources and Examples

**DOI:** 10.3389/fimmu.2019.02027

**Published:** 2019-08-27

**Authors:** Keith E. Garrison, Melanie R. Gubbels Bupp

**Affiliations:** ^1^Saint Mary's College of California, Moraga, CA, United States; ^2^Randolph-Macon College, Ashland, VA, United States

**Keywords:** lab, ELISA, flow cytometry, epitope prediction, pedagogy

## Abstract

Laboratory courses in immunology require a different skill set for their development than lecture courses. They vary widely in their form based on factors like institutional budget and class size, and also in the prioritization of learning goals centered around reinforcing lecture concepts and/or building fundamental skills in the field of immunology. Lab activities can come from a variety of sources including published research protocols, commercial kits, computer-based tools or simulations, and case studies. Each has their own strengths, which will be explored here. There are also important decisions to make about how students will report their data, and what level of guidance in interpreting data is best to enhance student learning and growth. Finally, methods like use of rubrics can help ensure fair and efficient grading, especially with skills-based learning goals. Periodic assessment is important to ensure that activities contribute effectively to student learning and to guide improvements to the lab course over time.

## Introduction

### Why Labs in Undergraduate Immunology Courses?

Lab courses can be expensive and time-consuming for the instructor—why should they be an integral part of any undergraduate immunology course? Well-designed multi-week lab experiments that involve students in experimental design, data analysis, and science-specific communication uniquely reinforce course content, increase student appreciation of the scientific method, and refine communication skills. Additionally, the lab component of immunology courses can expose students to key discipline-specific techniques (such as flow cytometry), which can give those students who might be interested in pursuing laboratory positions an advantage.

Recognizing that students with a wide variety of motivations and career goals will likely be present in any given undergraduate course, it is essential that laboratory activities reinforce content-focused course learning objectives (1). Student participation in labs that are focused on the cellular and humoral components of the immune system forces them to spend more time considering each cell type's unique contribution to the immune response. This understanding provides a strong foundation and can prompt a greater enthusiasm for and an improved understanding of the complete immune response.

### Eliminating Potential Roadblocks in the Early Phases of Lab Exercise Design

Clearly establishing the cost expectations for the lab within the context of department budgets is very important. This will determine a number of downstream variables such as use of kits, reagent availability, and staff support time. It is helpful to budget for the lab to cost at least 10% more than expected for unforeseen costs, especially the first time a lab is implemented. Misunderstandings around cost expectations are a major potential pitfall, especially for new faculty. A frank discussion with the department chair is warranted to establish clarity.

Instrumentation and facility availability are additional potential issues for the selection and design of lab exercises. Flow cytometry exercises are impractical without ready access to a flow cytometer. However, in some cases, access to instrumentation simply enhances an exercise. ELISA assays with visual readouts are more qualitative, whereas access to a plate reader makes them more quantitative. Activities involving cell culture require sufficient cell culture facility capacity to accommodate coursework. Higher level activities like this typically rely on smaller lab spaces designed for research. A lab may be more effective if students are split into smaller groups, which allows the instructor more time with students, but may reduce total lab hours for the course. Additionally, while students can be sub-divided into groups, the instructor cannot. Some activities require close supervision while others can be more independent.

Determining the appropriate level of guidance to offer is important at every phase of the exercise design. Advanced students may benefit from receiving manufacturer's protocols with minimal additional guidance to help them begin to adapt to future independent work in a lab setting, whereas detailed protocols, and analysis guidance may be appropriate for introductory or non-major's courses.

## Potential Sources of Material for Lab Exercises

### Adapting Protocols From Research Literature: Potential Issues

Work involving human samples is enticing to students with clinical interests, but it can involve its own set of regulatory challenges. Campus rules determine which human samples students may use. Even if students work with their own cells, a blood draw coordinated with the help of a qualified professional (nurse, phlebotomist, or other clinician), together with the processing of cells pose challenges. KG's immunology lab course has used an HLA sequencing protocol from the literature that was initially developed to work with the small amount of cellular DNA present in plasma samples (2). The sensitivity of the amplification protocol allowed the use of cells from cheek swabs as a source of genetic material when the protocol was adapted for HLA typing in the classroom (3). Materials for the classroom exercise included cheek swabs, DNA extraction kits (or chelex resin), PCR mix (beads or liquid), and primers. The products can be sequenced in house or commercially. As such, the cost of implementing this protocol could vary a bit, depending on choices made by each instructor. Although KG's institution does not have its own sequencing facilities, we were able to sequence samples for a reasonable cost at a local research institution. We also regularly perform PCR in multiple labs, so we were able to obtain master mix and primers for nominal costs. The published research protocol was developed to sequence all HLA Class I loci, but our classroom exercise only analyzed the HLA-B locus. Secretory IgA ELISA kits are also available to work with saliva samples (e.g., Abnova). These approaches eliminate the need for a blood draw, but the problem of safe handling of biological materials, and waste disposal still exists (see Supplementary Materials). Alternatively, students may work with cell lines or DNA, which can be purchased from ATCC (www.atcc.org). Lastly, it is important to consider the regulatory approvals that may be required, especially if any data collected from or about the students and their experiences in the lab course may be published. Institutional Review Board (IRB) approval may be necessary for research using human samples, even when the research is conducted in a classroom setting. Institutional Animal Care and Use Committee (IACUC) approval is needed for the use of vertebrate animals in the classroom, even if the uses are purely instructional. For tips on the safe handling of mice, consult Assessing the Health and Welfare of Laboratory Animals' website (http://www.ahwla.org.uk/site/tutorials/BVA/BVA05-Mouse/Mouse.html). Sources of specific protocols adapted for undergraduate labs include the Association for Biology Laboratory Education (http://www.ableweb.org/volumes/volume-38/), and CourseSource (https://www.coursesource.org/). Some journals emphasizing laboratory protocols for undergraduate education include CBE—Life Sciences Education and the Journal of Microbiology and Biology Education.

### Kits Developed for Classroom Use

Several manufacturers produce kits developed specifically for educational use. Kits are incorporated into the ELISA (Bio-Rad and NeoSci) and Western blot (Bio-Rad) units in KG's lab courses. Edvotek also provides kits to perform immunology experiments. Kits are usually set up to provide a defined number of student workstations or assays (12 workstations of 2–4 students for the Bio-Rad ELISA kit and 8 workstations for the BioRad Western blot kit; the Neo-Sci ELISA kit contains enough reagents for 40 students working individually). The potentially high cost per student (especially when compared to purchasing raw materials to prepare all the necessary reagents) may be offset with a reduction in in-house development time and resources dedicated to reagent preparation, validation, and quality control. The use of kits with an established track record of success also allows the instructor to focus on other aspects of the activity more closely tied to higher order learning goals for students, like data interpretation, and analysis. While kits may not always closely emulate the research lab experience, many clinical assays are kit-based. Students who go on to work in clinical or core labs may benefit from the experience they gain by following detailed protocols closely and carefully.

### Kits/Reagents for Flow Cytometry

Flow cytometers can be used in an undergraduate immunology course in a number of ways. A number of manufacturers offer an array of antibodies and kits that can help guide lab design (Table 1). As a way to reduce cost, students can work in small groups with a small number of samples and then the results from each group can be pooled for data analysis. Instructors wishing to incorporate flow cytometry may find it useful to first provide archived data for students to practice analyzing, as has been done in other undergraduate laboratory settings (4, 5).

**Table 1:** Examples of reagents and kits available for use with flow cytometers that may be useful in designing lab activities for undergraduate immunology courses.

**Topic**	**Suggested cell types**	**Reagent (Manufacturer, Cat. No)**	**Cost**	**Students supported by one kit**
Cell proliferation	Mouse T cells, B cells, Jurkat cells	CFSE-labeling (Tonbo, 13-0850-U500)	$23/500 μg	Quantity sufficient to label up to 10 × 10^6^ cells on 20 separate occasions; depending on experiment details, quantity sufficient for 60 groups of students
Phagocytosis	Mouse bone-marrow derived macrophages, THP-1 cells	FITC-labeled latex beads for phagocytosis assays (Cayman Chemical, 500290)	$250/750 samples	One kit sufficient for 4 groups of students
Apoptosis	Mouse primary cells, Jurkat cells	Caspase-3/7 Fluorescence Assay Kit (Cayman Chemical, 10009135)	$198/96 wells	One kit sufficient for 6 groups of students
Apoptotic cell clean-up/Lupus	THP-1 cells + any cell as “apoptotic bait”	Efferocytosis Assay Kit (Cayman Chemical, 601770)	$295/kit	Quantity sufficient to label up to 10 × 10^7^ effector cells and 2 × 10^8^ bait cells; depending on experiment details, could support 15 groups of students
Cell viability	Any cell (primary or cell line)	PE Annexin V Apoptosis Detection Kit with 7-AAD (Tonbo, 50-6410-KIT)	$195/100 tests	Depending on experiment details, one kit sufficient for 10 groups of students
Cytokine production	Mouse T or B cells, Jurkat cells	Intracellular cytokine staining kit (protocol is for mouse, but can also purchase kit for use with Jurkat cells) (BD Biosciences, 554715)	$265/250 tests	Depending on experiment details, one kit sufficient for 20 groups of students
Identifying regulatory T cells	Mouse splenocytes	Foxp3 Transcription factor staining kit (ThermoFisher, 00-5523-00)	$165/kit	Depending on experiment details, one kit sufficient for 10 groups of students

### Computer-Based Tools/Simulations

The use of computer-based tools or simulations may permit students to explore areas where a program lacks instrumentation or resources to explore with a hands-on lab activity. In KG's lab course, students learn to use an online epitope prediction algorithm NetCTL (http://www.cbs.dtu.dk/services/NetCTL/). These epitope prediction algorithms are useful to help select a set of peptides for measuring immune responses when the potential number of candidate epitope peptides is large and the budget for peptide synthesis is limited. The lab activity emulates the validation experiments done for this algorithm (6). Other epitope prediction programs are reviewed and summarized in (7, 8). To guide students to identify metrics of success in advance, posing specific analytical questions in the pre-lab materials is helpful (see Supplementary Materials).

### Case Studies

Case studies are a useful tool in the immunology lab course. Depending on the case study selected, teaching of additional clinical or public health related background material may be needed. Some of the publishers of immunology textbooks produce companion volumes of case studies with additional tools to help incorporate those case studies into an immunology course (e.g., Norton & Co.) Many web-based resources are also available. The National Center for Case Study Teaching in Science provides resources (http://sciencecases.lib.buffalo.edu/cs/). This site includes an interesting case study about the analysis of flow cytometry data (http://sciencecases.lib.buffalo.edu/cs/files/flow_cytometry.pdf) that could be especially helpful for instructors who want to teach the technique but are at an institution that lacks access to an instrument. KG uses a case study developed in the training program for the Epidemic Intelligence Service of the Centers for Disease Control (EIS) (https://www.cdc.gov/eis/casestudies/XscreeningHIV.student.871-703.pdf). This activity requires teaching some additional background on determining the predictive power of clinical assays, but this background is mostly developed in the context of the activity. Use of this activity also introduces students to a component of the US Public Health infrastructure that may offer future training or career opportunities for them.

## Examples of Inquiry-driven Lab Modules

We present here three Immunology lab modules targeted to upper-level Biology majors. All of the modules span multiple weeks, are inquiry-driven, and gradually involve students in experimental design.

In module one, students induce a sterile inflammatory response by injecting 1 ml of 6% thioglycollate into the peritoneal cavity of mice and follow the immune response over time (3 mice each at 0, 2, 24, and 48 h). Cellular changes in the blood are analysed using blood differentials and in the peritoneal cavity using flow cytometry. Detailed instructor notes and student handouts are provided as Supplementary Materials.

The second module is broadly focused on communication between the innate and adaptive immune response; students analyze the effect of a particular substance on the ability of cultured macrophages to phagocytose latex beads. Students are first introduced to cell culture and are assigned a culture of THP-1 cells to take care of for the week. Next, each group brainstorms a list of substances/compounds that they hypothesize might affect phagocytosis such as garlic extract, honey, particular vitamins/minerals, etc. The class agrees on a substance and designs an experiment to test the effect of the chosen substance on the ability of THP-1 cells to phagocytose FITC-coated beads. Students consult the literature to determine methods of delivery, concentration, and the timing of application of the substance to the culture. Note that if a flow cytometer is not available, an instructor may instead use a fluorescent microscope to assess phagocytosis. Detailed instructor notes and student handouts are provided as Supplementary Materials.

The last module builds on the skills the students have practiced in the first two modules. The instructor introduces 1-3 key questions that students choose from. Groups of students present their experimental design ideas and the class votes on one research project (with feedback from the instructor about what is feasible given the number of lab sessions and resources available). In the past, students have chosen to determine if antibiotic exposure influences the isotype or quantity of antibody elicited by vaccination against OVA or KLH antigens using ELISAs. Importantly, to implement this option, instructors must also account for the time needed to order supplies.

## Modular and Multi-disciplinary Approaches to Topic Areas in Lab Courses

In KG's ELISA labs, the technique is approached from several different directions. The qualitative nature of the technique (positive/negative test for an antibody response) is explored using the Neo-Sci kit (Neo/Sci Corporation) with a visual readout. The quantitative nature of the assay is explored using the Bio-Rad kit with a readout requiring a spectrophotometric plate reader. Lastly, the public health implications of the assay are explored using the case study activity developed by EIS.

The objective of the first subset of labs is to teach students about (1) the diagnostic significance of ELISA in clinical settings and (2) the limitations of ELISA tests in clinical settings. It is also an opportunity to introduce students to the challenges involved in discussing scientific results as “clinicians” in understandable terms with other students who role-play as “patients.”

The quantitative section involves students generating a standard curve and measuring the concentration of the target in two unknown samples. This activity allows for the assessment of two interlinked outcomes. The first outcome relates to the precision of student pipet technique, which can be evaluated using the reproducibility of replicates in the standard curve. This offers a rare opportunity to make an objective assessment of a skills-based learning goal based on accurate and reproducible pipetting of small volumes. The second is the ability to estimate an unknown quantity using a standard curve. Students at Saint Mary's College of California complete a lab activity in their introductory biology course series involving total protein quantitation using Bradford reagent. With this background knowledge, upper-division students can evaluate the additional advantages of using a protein-specific antibody to quantify a single target protein of interest.

The case study activity supports the exploration of public health uses of ELISA assays. Because this is a more advanced application of the assay, it is done towards the end of unit dedicated to the ELISA. Students are already familiar with the technical aspects of the assay. Because they have seen the effects of pipetting precision on the outcomes of the standard curve and determination of unknowns, they are better able to identify potential technical faults in the assay. This activity helps students to see the changes in predictive value positive and negative in populations with greater or lesser prevalence of a tested antigen (in this case, HIV).

## Student Presentation of Lab Data

The classic lab report is a commonly used method for instructors to assess student performance in lab. Alternatively, students can make small group or class presentations of their data and analysis, an approach that can increase student accountability for data quality. Additionally, as a way of differentiating the presentations from each other, groups of students can be assigned different discussion questions to incorporate into their presentations (see instructor notes for projects 1 and 2 in Supplementary Materials for examples).

Posters are a good way to convey results succinctly with an emphasis on visual impact and clear figure construction. Mock manuscripts with all the sections contained in a typical peer reviewed paper are another approach that can work especially well with advanced students. Ideally, teaching students to write mock manuscripts would begin in the introductory course and be reinforced throughout the curriculum. Posters and presentations can include a peer evaluation component.

## Assessment of the Value of Lab Activity

Designing labs around predefined learning goals enables multi-part lab activities with time for reflecting on progress and interpretation of the data. Sometimes the core principle or skill behind the goal can be taught most effectively with a simpler technique that is easier for students to grasp and more likely to generate meaningful data in their hands. However, teaching students current techniques and valued laboratory skills can translate into jobs for students upon graduation. Evaluating skills and behavior-based learning goals is an interesting area of assessment research, especially in training and evaluating medical residents [(9); American College of Physicians, accessed 2019^1^]. Some of these goals for medical resident evaluation programs are adaptable to the identification and measurement of skills-based learning goals at the undergraduate level.

Student impacts are measurable in a number of ways. Changes in student knowledge can be measured through pre- and post-testing around any given lab activity. Current student reflections and feedback can be a valuable source of information about the impacts of activities. Periodic assessment by former students who go on to graduate or professional study or work in the biotechnology industry can provide valuable feedback on the relevance of current lab activities and be potential sources of new lab activity ideas. Lastly, it is important to incorporate mechanisms to respond to constructive feedback and update activities as time progresses.

## Author Contributions

KG contributed experience in running computer simulation, ELISA, and case study labs and wrote these portions of the manuscript. MG contributed experience in running flow cytometry labs and wrote these potions of the manuscript. Both authors contributed collaboratively to other portions of the manuscript.

### Conflict of Interest Statement

The authors declare that the research was conducted in the absence of any commercial or financial relationships that could be construed as a potential conflict of interest.

## Footnotes

^1^American College of Physicians. Available online at: https://www.acponline.org/about-acp/about-internal-medicine/career-paths/residency-career-counseling/preparing-for-internal-medicine-board-certification/the-board-certification-process/about-the-residency-performance-and-competency-evaluation-process (accessed 2019).
