# Supplementary material for: Setting Up an Undergraduate Immunology Lab: Resources and Examples
Source: Front Immunol. 2019 Aug 27;10:2027. doi: 10.3389/fimmu.2019.02027 (PMC6718614; doi:10.3389/fimmu.2019.02027)
Supplement: Supplementary file 1 [file Data_Sheet_1.PDF]

BIOL 139: Immunology
Garrison Fall 2018

The central question for the lab:

How effective are epitope prediction algorithms at predicting real CTL epitopes found in pathogens?

For your pre-lab, take notes in your notebook on the pre-lab explorations that you do to familiarize yourself with these online tools. Do <u>not</u> simply print and paste this document into your notebook. You can paste the HIV genome maps on the last page into your notebook, if you wish.

Here is the webpage for the 'NetCTL' algorithm we will be testing:
http://www.cbs.dtu.dk/services/NetCTL/

Please visit the site and review the factors that this algorithm computes into its epitope prediction process along with the components of the MHC Class I presentation pathway. **<u>Put information about the variables that the algorithm uses into your notebook.</u>** Are any factors missing from consideration by the algorithm? If so, can you think of why these factors might be difficult or impossible to predict?

Not all pathogens have epitope maps because they require a lot of information and study to compile. Researchers are very interested in the immune response to HIV, so numerous scientists have worked very hard to produce these maps. Here are the epitope maps for HIV proteins:

http://www.hiv.lanl.gov/content/immunology/maps/maps.html

Make sure you understand how to read these maps. If the prediction algorithm focuses on MHC Class I presentation, which epitope maps will be our focus for lab?

The maps will be our method of testing the effectiveness of the algorithm, because they give us something to measure the efficiency of prediction. The maps come from a collation of data from numerous studies using cell samples from HIV+ individuals to identify immune responses against the virus.

HLA (or MHC) types are genetic variations found in each of us that allow our immune systems to present different parts of pathogen proteins to our T cells. There are so many individual HLA types that it is useful to identify groups of HLA types that share common features, called HLA supertypes. Maps generally show immune responses associated with an HLA type, whereas the prediction algorithms work with HLA supertypes. Therefore, you need to be able to interconvert between HLA types and supertypes. Here is a list of HLA types that are included in each supertype. You may need to use this list as a reference during lab.

http://www.hiv.lanl.gov/content/immunology/motif_scan/supertype2.html

Here are the relevant HIV proteins:

http://www.ncbi.nlm.nih.gov/nuccore/nc_001802.1

We are using the HXB-2 sequence because it is the nucleotide and amino acid numbering standard for HIV-1. Numbering in a consistent way with HIV-1 is challenging, considering the amount of viral sequence variability within and between individuals.

Initially, we will focus on Gag, Pol and Env proteins. Time permitting, we can move on to accessory proteins like Nef and Vif. On the next page, you can find a map of the various proteins in the HIV genome.

We should also consider what metrics we will use, and some questions associated with those metrics:

- What threshold does the algorithm apply to identify and assign an epitope?
- How frequently does the algorithm identify an epitope correctly (i.e. T cells recognize the epitope in samples from real people)? How will you recognize this in your data?
- How frequently does the algorithm identify an epitope when one does not exist? How will you recognize this in your data?
- How frequently does it fail to identify an epitope when one does exist? How will you recognize this in your data?

Be sure to answer these questions in your lab notebook. If you have trouble answering them, come to see me in office hours before lab.

If you have extra time in lab, you can test these additional parameters:
1. Compare NetCTL to HLA/MHC binding prediction alone? Does NetCTL perform better than HLA/MHC binding prediction alone at identifying real epitopes?
2. Manipulate the adjustable algorithm parameters. Do any improve the ability to predict epitopes, especially altering the score min. threshold?

Genome Maps

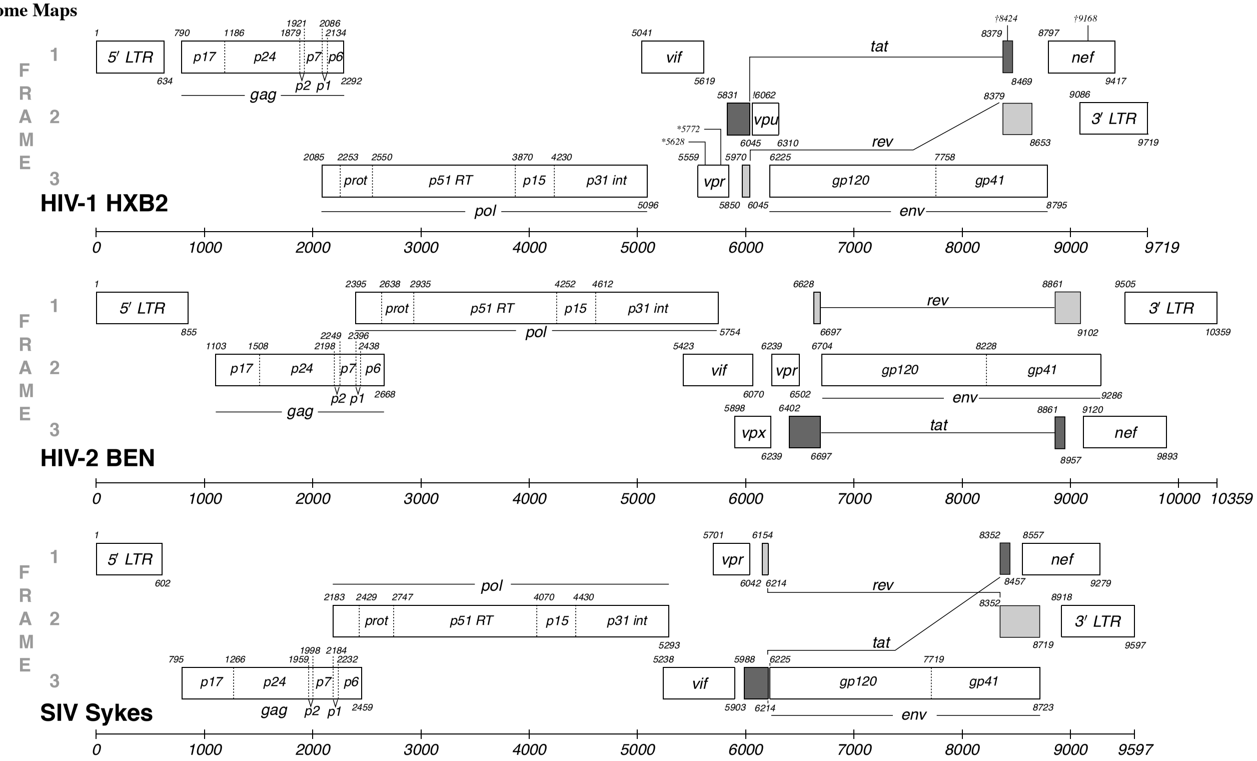

Landmarks of the HIV-1, HIV-2, and SIV genomes. The gene start, indicated by the small number in the upper left corner of each rectangle normally records the position of the `a` in the `atg` start codon for that gene while the number in the lower right records the last position of the stop codon. For *pol*, the start is taken to be the first `t` in the sequence `tttttag` which forms part of the stem loop that potentiates ribosomal slippage on the RNA and a resulting –1 frameshift and the translation of the gag-pol polyprotein. The *tat* and *rev* spliced exons are shown as shaded rectangles. In HXB2, **5628* and **5772* mark positions of frameshifts in the `vpr` gene; !6062 indicates a defective `acg` start codon in *vpu*; *†8424*, and *†9168* mark premature stop codons in *tat* and *nef*. See Korber et al., Numbering Positions in HIV Relative to HXB2CG, in *Human Retroviruses and AIDS, 1998* p. 102. Available from `http://hiv-web.lanl.gov/HTML/reviews/HXB2.html`.
