# Supplementary material for: Setting Up an Undergraduate Immunology Lab: Resources and Examples
Source: Front Immunol. 2019 Aug 27;10:2027. doi: 10.3389/fimmu.2019.02027 (PMC6718614; doi:10.3389/fimmu.2019.02027)
Supplement: Supplementary file 2 [file Data_Sheet_2.PDF]

Project 1: Effect of Peritonitis on leukocyte trafficking Labs 1-4

Background:

The first half of project 1 involves performing a **differential blood count** with mouse blood obtained before and after **peritonitis** (inflammation of the peritoneum, the tissue that lines the wall of the abdomen and covers the abdominal organs) is induced. The second half of project 1 is focused on monitoring the cellular dynamics of the intraperitoneal inflammation itself, utilizing **flow cytometry**.

Thioglycollate-Induced Peritonitis: Recruitment of leukocytes from the circulation and their subsequent influx into the sites of inflammation is critical for host defense and wound healing. This is a multistep process, which is regulated, in part, by adhesion molecules and chemokines that are upregulated during inflammation. An intra-peritoneal injection of thioglycollate generates local inflammation and initiates the migration of inflammatory cells to the site of inflammation. Thus, thioglycollate-induced peritonitis in mice mimics an acute inflammatory response in the peritoneum.

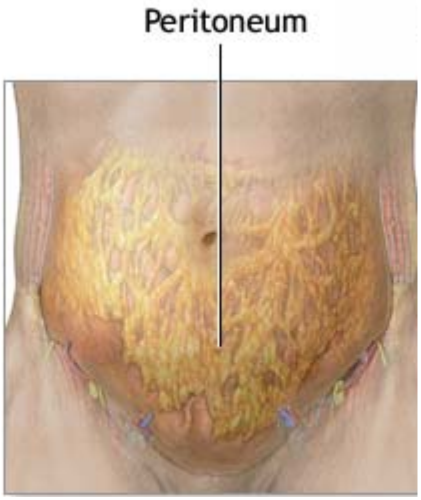

Reading:

Exercise 11-1: Differential Blood Cell Count from Leboffe and Pierce's "Microbiology Laboratory Theory and Application" 4th edition.

Chapter 1 from Parham's "The Immune System", [browse pages to find examples of H & E stained immune cells]

Lab Session 1

Blood Differentials

1. Label microscope slides with one group member's initials and "C" for control + the number of the mouse sampled.
2. Anesthetize mice (one at a time) with isoflurane.
3. Place mice under heat lamp until tail vein is visible (about 1 min). CAUTION: Mice can overheat and go into shock, so take care not to place them under the heat lamp for excessive lengths of time.
4. Wipe tail vein with ethanol (to sterilize) and gently make a shallow incision with a razor. Allow one drop of blood to land on microscope slide. Apply pressure to the tail until bleeding stops.
5. Meanwhile, your partner should obtain a second microscope slide and drag the blood drop with the corner of the slide so that a smear results. NOTE: The objective is to create a single layer of cells on the slide. Once you have smeared the blood do not smear it again.

6. Allow blood to dry and perform staining:

   a. Dip slide in Fixative Solution 5 times, 1 sec each time. Allow excess to drain onto a paper towel

   b. Dip slide in Solution I 5 times, 1 sec each time. Allow excess to drain onto a paper towel

   c. Dip slide in Solution II 5 times, 1 sec each time. Allow excess to drain onto a paper towel

   d. Rinse slide with deionized water

7. Allow slides to dry and visualize them under the 40X

8. Look over the slide until you have identified at least 5 lymphocytes, monocytes, and neutrophils.

9. Now examine the slide under oil immersion (100X) using a drop of oil.

10. Follow a systemic path on the slide (see figure 10-6 of handout) and record the numbers of leukocytes observed on your worksheet until you have identified and recorded 100 total cells. These observations indicate the normal percentages of specific leukocytes in control mice and will be used to compare blood differentials of mice undergoing peritoneal inflammation.

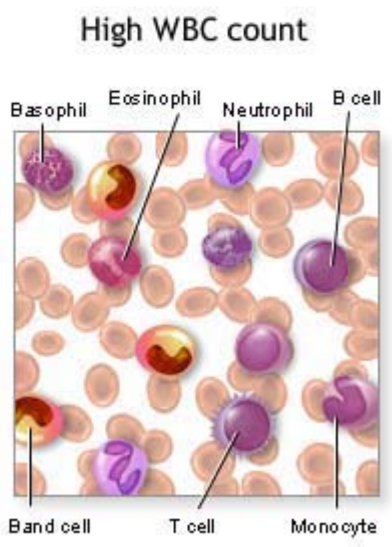

(Picture taken from http://www.nytimes.com/slideshow/2007/08/01/health/100151Whitebloodcellcountseries_index.html --access site to learn more about how differentials are used in diagnosis.)

Project 1: Effect of Peritonitis on leukocyte trafficking Labs 1-4
Lab Session 2

Blood Differentials

1. Perform a blood differential on mice with ongoing peritonitis, following the same protocol as last week. Be sure to label the slides as “d2” for day 2, “24h” for the 24 hours, and “2h” for the 2 hour timepoint.

2. Enter data in lab notebook.

Thioglycollate-Induced Peritonitis – Harvesting cellular infiltrate

1. Euthanize mice (instructor will do this). Wet abdomen with 70% ethanol to sterilize the area. Make a midline incision---be careful to cut through the skin layer only and NOT the peritoneum! Retract the abdominal skin to expose the intact peritoneal wall.

2. Inject 7 mls of PBS + 1 ml of air into the peritoneal cavity with a syringe and 23G needle, beveled end of the needle facing up. NOTE: It is very important to puncture the peritoneum only once! After you have injected the fluid AND the air, use a tweezers to close the hole the needle created as you remove the needle. Continuing to seal the hole with the tweezers, rotate the mouse side to side.

3. Lay the mouse on its side. Ask your partner to insert the needle beveled end UP into the pocket of air and slowly withdraw fluid, taking care not to puncture the intestines or other organs. NOTE: Keep needle away from fat deposits, as fat will clog the needle—keeping the beveled end of the needle UP will help with this.

4. Transfer the fluid to a 15 ml conical tube and centrifuge for 5 minutes at 1500 rpms.

5. **While cells are spinning**, label three (or four) 1.5 ml tubes with the mouse IDs (i.e. 2hr-1, 2hr-2, 2hr-3, etc.) that your group is taking care of. You will transfer cells to this tube for staining purposes.

6. Label ANOTHER three (or four) 1.5 ml tubes with the mouse IDs (i.e. 2hr-1, 2hr-2, 2hr-3, etc.) that your group is taking care of, and add “-C” on the end of each label. You will transfer cells to this tube to count them.

7. Dump the supernatant into the sink, taking care not to disturb the cells that have pelleted to the bottom.

8. Gently resuspend the pellet in 1 ml (for 2 hr or 26hr mice) or 100 µl (for 0 hr controls) of FACS Staining Solution using a pipette. **RECORD the volume of FACS Staining Solution used on page 4, step #1.**

9. Transfer 10 ul from each 15-ml tube to the appropriately labeled 1.5 ml tubes labeled with mouse ID-C (for counting). [These are the cells you will count.]

10. Transfer 100 µl (using a pipette) to labeled eppendorf tube and spin in tabletop centrifuges at LOW SPEED (1.5 on the dial) for 5 minutes. [These are the cells you will stain.]

11. Remove the supernatant with a p100 dialed to 80ul, taking care not to disturb the cells that have pelleted to the bottom.

12. Gently flick the pellet to resuspend and then add 10 µl of diluted Fc-block (CD16/32, diluted 1:5 in FACS Staining Buffer).

13. Add 10 ul of "Antibody mix" to each 1.5 ml tube of cells labeled with the mouse ID. The antibody mix tube contains antibodies to the proteins, CD3, CD19=PerCPCy5.5, Ly6G, and CD11b. Each antibody is conjugated to a fluorescent tag. The fluorescent tags are light-sensitive, so take care to keep them in the dark as much as possible (wrap eppendorf tubes with aluminum foil).

    Your mix contains the following antibodies/fluorescent tags:
    a. CD3 – APC
    b. CD19 – PerCP-Cy5.5
    c. Ly6C/G – FITC
    d. CD11b – PE

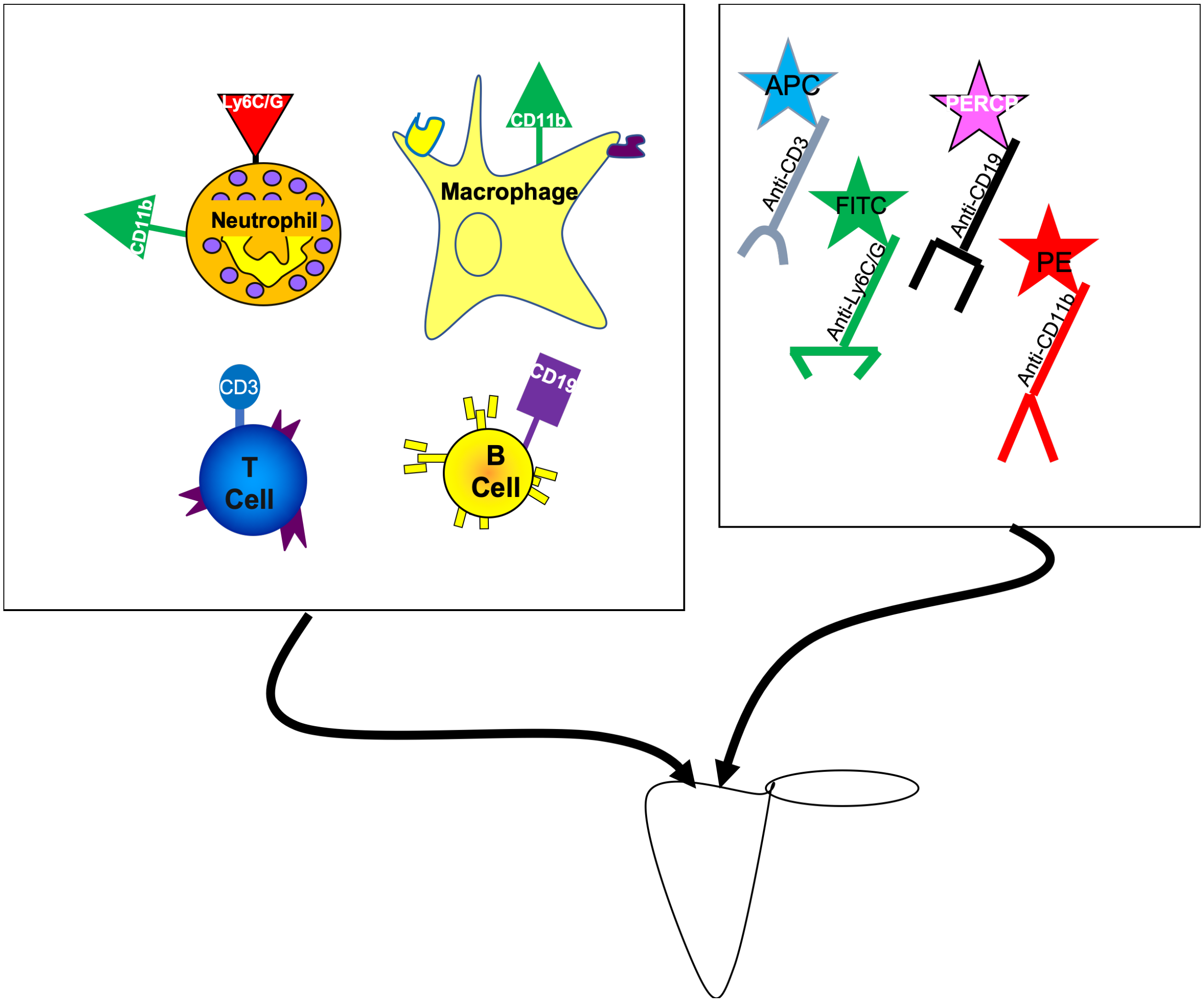

14. The cells are now staining. Allow them to stain for 30 minutes (if time allows). Use this time to stain and analyze blood smears and count peritoneal cells (see session 1 handout and "Counting Cells" protocol below).

15. After 30 minutes, wash out excess (unbound) antibody by adding 1 ml of 1X PBS to stained cells. Spin AT LOW speed (1.5 on the dial) in table top centrifuge for 5 minutes.

16. Dump the supernatant into the sink, taking care not to disturb the cells that have pelleted to the bottom. Add 200 µl of 4% Paraformaldehyde to fix the cells for later acquisition on the flow cytometer (next week). Store in the refrigerator.

COUNTING CELLS

1. Record volume that cells were resuspended in step #8. _________________
2. Mix 10 µl of resuspended cells with 10 µl of Trypan Blue. Note, this is diluting your concentration of cells in half. (10/20…)
3. Carefully lay the glass cover slip onto the hemocytometer. Pipette 10 µl of your cell/Trypan Blue mix into the hemocytometer chamber. (see diagram below)

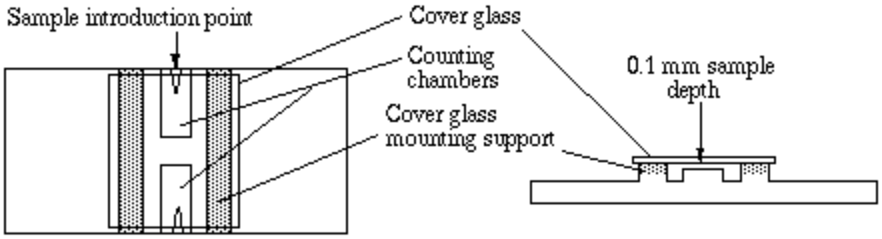

4. Look at the cells in the hemocytometer under the microscope—10X is the best objective to use. Be very careful not to crush the cover slip with the objective—hemocytometers are considerably thicker than microscope slides!
5. Using a hand tally, count the number of cells (don't count blue ones, they are dead cells) in the **center 5 x 5 grid** (see diagram on right).

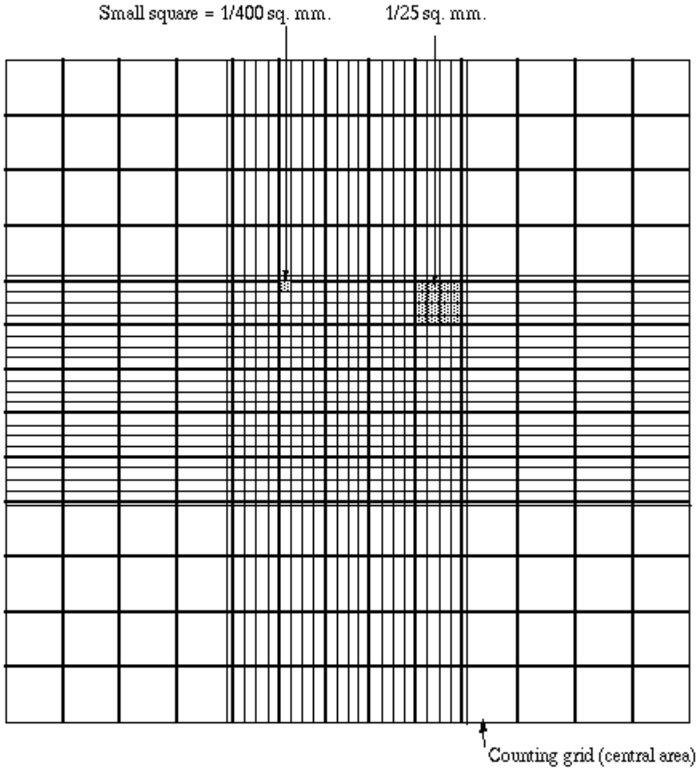

6. Record the number of cells counted here: ____________________.

7. Use the following equation to determine the total number of cells you isolated from the peritoneum:

   (# of cells counted) X (trypan blue dilution factor) X 10^4 X (original volume in mls)

   OR

   (#6) * 2 * 10^4 * (#1)

   EXAMPLE:

   --I resuspended my cells in 1 ml of FACS Staining Buffer and the diluted 10 µl of that suspension with 10 µl of Trypan Blue. I counted 63 cells in the hemocytometer. Therefore:

   63 * 2 * 10^4 * 1 ml = 124 x 10^4 = 1.2 x 10^6

   I isolated 1.2 million cells from this mouse's peritoneal cavity. In immunology, it is customary to talk about cell concentrations in millions of cells, or 10^6.

8. Count the number of cells harvested from each mouse using this method. Be sure to record your data for ALL mice. **NOTE: You will want to be able to correlate the staining results with the cell counts harvested, so make sure the labels on the eppendorf tubes match the labels in your notes.**

Project 1: Effect of Peritonitis on leukocyte trafficking Labs 1-4

Lab Session 3

1. Acquire cells on Cytometer.

2. Meanwhile, examine blood smears, exchange data on blood differentials and cell counts from session 2.

3. Enter data into excel and graph results. You should have a graph that exhibits each of the following:
   a. the average *percentages* of each type of white blood cell in blood smears obtained from control, 2 hrs, and 28 hours mice.
   b. the average *percentages* of each type of cell (T cells, B cells, Neutrophils, and Macrophages) obtained from the peritoneal cavity of control, 2 hrs, and 28 hours mice.
   c. the average *total number* of cells obtained from the peritoneal cavity of control, 2 hrs, and 28 hours mice.
   d. the average *total number* of each type of cell (T cells, B cells, Neutrophils, and Macrophages) obtained from the peritoneal cavity of control, 2 hrs, and 28 hours mice.

   Graphs should be complete with error bars, statistics, and figure legends.

4. Perform appropriate statistical analysis.

5. Create Powerpoint presentation (due next week) or short report (due in two weeks) as assigned.

6. Perform literature searches as appropriate.

7. When percentages of each cell type have been obtained from the cytometer, multiply the percentages of T cells, B cells, Macrophages, and Neutrophils by the total number of peritoneal cells for each sample to determine the total number of each cell type present in the peritoneal cavity.

EXAMPLE: Let's imagine you calculated (using a hemocytometer and microscope) that you isolated 3 million (**3 x 10^6) cells** from the peritoneal cavity of a control mouse (received no thioglycollate injection). You then obtain the FACS plot shown below after acquiring the stained cells from the same control mouse on the flow cytometer.

Macrophages express CD11b, but not Ly6G/C. Therefore, the FACS plot indicates that **73%** of the peritoneal cells in this particular sample are macrophages. Neutrophils express both CD11b and Ly6G/C, so the data indicate that there are no neutrophils present in this particular sample.

To calculate how many total macrophages were present in the peritoneal cavity, multiply 0.73 and 3 x 10^6. There were 2.2 x 10^6 macrophages present in the control mouse's peritoneal cavity.

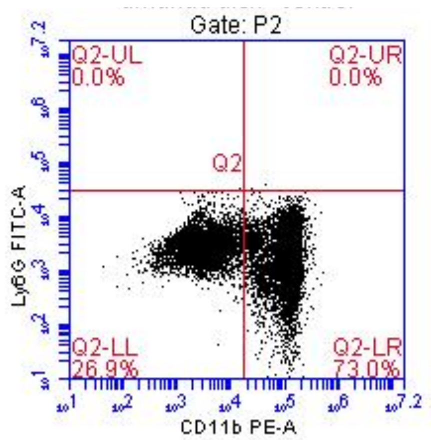

Project 2: Phagocytosis Labs 4-7

Lab Session 4: Cell Culture

PURPOSE:

1. Culture human monocytes (the precursor cell to macrophages) and then use them to assess the effect *differentiation* (inducing them to become macrophages) has on the ability of these cells to phagocytose latex beads.

2. Learn cell culture techniques

THP-1 cells are monocytes originally obtained from a human and are now continually grown by scientists in the lab.

Important notes:

Each group will receive one flask of THP-1 cells of an unknown concentration. Your group must determine the concentration of the cells. Using that information, you will then ensure that the cells have an adequate amount of "food" (media) today.

At least one member of each group must "feed" the cells on Fri.

Another group member must transfer a particular number of cells to a 24 well plate on Monday (in preparation for our experiment on Tuesday).

Next week, we will incubate our cells with FITC-labeled latex beads and assess phagocytosis.

GENERAL STERILE CELL CULTURE TECHNIQUE:

1. Wipe your work area and hands with 70% ethanol before starting.

2. Never uncover a sterile flask, bottle, petri dish, etc., until the instant you are ready to use it. Return the cover as soon as you are finished. Never leave it open to the environment.

3. When removing the cap from a bottle, flask, etc., do not place the cap with the open end upright on the lab bench. Do not hold the opening straight up into the air. If possible, tilt the container so that any falling microorganisms fall onto the lip.

4. Do not draw from different bottles with the same pipette. Because such a pipette has been exposed; the chance for contamination is too great; use a sterile pipette for each bottle -- especially when pipetting media.

5. Techniques should be performed as rapidly as possible to minimize contamination.

PROTOCOL FOR TODAY & FRIDAY:

1. Place a bottle of THP-1 media in the 37 degree water bath.
2. Working in the hood (be sure you sprayed the flask and your hands before placing them in the hood), thoroughly resuspend the cells in your flask by pipetting them up and down with a transfer pipette.
3. Remove 10 ul of the resuspended cells from the flask into a clean eppendorf tube.
4. Working outside of the hood now, add 10 ul of Trypan Blue to the 10 ul of cells. Mix thoroughly. (Note, this is a 2 fold dilution of your cells [10/20=2]).
5. Carefully lay the glass cover slip onto the hemocytometer. Pipette 10 µl of your cell/Trypan Blue mix into the hemocytometer chamber.
6. Look at the cells in the hemocytometer under the microscope at 10X
7. Using a hand tally, count the number of LIVE cells (not blue) in the **center** 5 x 5 grid
8. Record the number of LIVE cells counted here: _____________________.
9. Make note of the volume of media contained in your flask of cells:________________.
10. Use the following equation to determine the total number of LIVE cells present in your flask:

    (# of cells counted) X (trypan blue dilution factor) X vol (#8) X 10^4

    OR

    (#LIVE cells) * 2 * (#8) * 10^4 = _______________________

    EXAMPLE: I counted 63 cells in the hemocytometer. My flask had 5 mls in it. Therefore the flask contains 6.3 million cells.

    $63 * 2 * 5 * 10^4 = 6.3 \times 10^6$

11. Cells have enough food when they are cultured at 0.3×10^6 cells/ml. Use the equation below to adjust the volume of media so that your flask of cells has the correct amount of "food" or media.

    (total # of live cells)/0.3×10^6 = total FINAL volume of media

    OR (using the example above)

    $6.3 \times 10^6 / 0.3 \times 10^6$ = 21 mls of media

    Since our cells (in the example) are already in 5 mls of media, we need to add 16 mls of additional media to our cells (21 mls – 5 mls = 16 mls).

12. Retrieve the (now warm) bottle of media from the waterbath. Spray it and your hands with ethanol and place it in the hood.

13. Add 16 mls of media to your flask.

PROTOCOL FOR MONDAY:

1. Place a bottle of THP-1 media in the 37 degree water bath.

2. Working in the hood (be sure you sprayed the flask and your hands before placing them in the hood), thoroughly resuspend the cells in your flask by pipetting them up and down with a transfer pipette.

3. Remove 10 ul of the resuspended cells from the flask into a clean eppendorf tube.

4. Working outside of the hood now, add 10 ul of Trypan Blue to the 10 ul of cells. Mix thoroughly. (Note, this is a 2 fold dilution of your cells [10/20=2]).

5. Carefully lay the glass cover slip onto the hemocytometer. Pipette 10 µl of your cell/Trypan Blue mix into the hemocytometer chamber.

6. Look at the cells in the hemocytometer under the microscope at 10X

7. Using a hand tally, count the number of LIVE cells (not blue) in the **center** 5 x 5 grid

8. Record the number of LIVE cells counted here: ______________________.

9. Use the following equation to determine the concentration of LIVE cells present in your flask:

   (# of cells counted) X (trypan blue dilution factor) X 10^4

   OR

   (#LIVE cells) * 2 * 10^4 = ________________________

   EXAMPLE: I counted 63 cells in the hemocytometer. Therefore the concentration of cells in my flask is 1.2 million cells/ml .

   $63 * 2 * 10^4 = 1.2 \times 10^6$ cells/ml

10. Use the following equation to determine what volume of cells you must remove from the flask to obtain 6 million cells.

   6×10^6 /cell concentration (#9) = volume needed

   EXAMPLE (from above): $6 \times 10^6 / 1.2 \times 10^6 = 5$ mls

11. In the hood (using sterile techniques) thoroughly resuspend the cells in your flask by pipetting them up and down with a serological pipette. Remove the volume you calculated in #10 using a serological pipette and place it in a 15 ml conical tube.

12. Adjust the volume of media in the 15 ml conical tube to 12 mls.

    EXAMPLE: There are 5 mls in the 15 ml conical tube. 12 mls-5 mls = 7 mls, so I need to add 7 mls of warm media to the 15 ml conical tube.

    After this adjustment, the cells are at a 0.5 x 10^6 cells/ml concentration.

13. Thoroughly resuspend the cells in the 15 ml conical tube by gently inverting it up and down several times (be sure the lid is screwed tight). Then, pipette 1 ml of resuspended cells into 6 wells of a 24 well plate (see diagram below).

14. Add 1 ul of PMA to the appropriate wells (see diagram below). (PMA is a differentiation reagent that will induce the monocytes to become macrophages.)

15. Add XXX of reagent of student's choice (to be defined) to the appropriate wells.

16. Place the 24-well plate in the incubator.

17. Remove all trash from the hood, clean the hood with ethanol, place your flask of cells in the sink and add about 10 mls of bleach.

	1	2	3	4	5	6
A	THP-1 cells	THP-1 cells + PMA	THP-1 cells + FITC latex beads	THP-1 cells + PMA + FITC latex beads	THP-1 cells + student choice	THP-1 cells + student choice + FITC latex beads
B						
C						
D						

Lab Session 5: Phagocytosis Assay

Today, we will incubate our cells with FITC-labeled latex beads and assess their ability to phagocytose!

Each group should have a 24 well plate in the incubator. At this point, the THP-1 cells are in wells A1-A6, PMA was added to wells A2, A4, and A6, and Vitamin C (Ascorbic acid) was added to wells A5 and A6.

PMA (Phorbol 12-Myristate 13-Acetate) is a small molecule that binds to and activates protein kinase C. It causes an extremely wide range of effects in different cells and tissues. It induces monocytes to differentiate into macrophages.

NOTE: Monocytes exist in cell suspensions (they do NOT adhere to the plate). However, macrophages adhere to tissue culture plates.

	1	2	3	4	5	6
A	THP-1 cells	THP-1 cells + PMA	THP-1 cells + FITC latex beads	THP-1 cells + PMA + FITC latex beads	THP-1 cells + FITC latex beads + Vitamin C (Ascorbic acid)	THP-1 cells + PMA + FITC latex beads + Vitamin C (Ascorbic acid)
B						
C						
D						

TODAY'S PROTOCOL:

1. Add 50 ul of FITC-latex beads to the appropriate wells (A3, A4, A5, and A6).
2. Incubate for 30 min at 37 degrees.
3. During the incubation, label 4 FACS tubes 1-6.

   Note: We will also plan for how to efficiently acquire each groups cell's on the flow cytometer.
4. At end of incubation transfer cells into labeled FACS tubes using a transfer pipet.
5. To remove any cells that have adhered to the culture plate, add 80 ul of trypsin to each well for

   NO MORE than 1 minute at room temp.
6. After 1 minute, add 1 ml of 1X PBS.

7. Transfer contents of well to appropriate FACS tube (combine with transferred contents from step 4).
8. Centrifuge 5 min 400 x g at room temp
9. Aspirate supernatant
10. Add 50 ul of ice cold diluted trypan blue (vortex at low power)
11. Add 1 ml of Assay Buffer and vortex (at low power)
12. Centrifuge 5 min 400 x g at room temp
13. Add 1 ml of Assay Buffer and vortex (at low power)
14. Centrifuge 5 min 400 x g at room temp
15. Add 500 ul of assay buffer to each tube, vortex (at low power)
16. Place cells on ice and acquire by flow cytometry immediately.
17. Class data will be compiled and posted to analyze next week.
