# Supplementary material for: Setting Up an Undergraduate Immunology Lab: Resources and Examples
Source: Front Immunol. 2019 Aug 27;10:2027. doi: 10.3389/fimmu.2019.02027 (PMC6718614; doi:10.3389/fimmu.2019.02027)
Supplement: Supplementary file 3 [file Data_Sheet_3.PDF]

Supplementary Methods

Biohazard handling guidelines

Students should be reminded to wear disposable gloves and other appropriate PPE at all times. Students are sometimes challenged to work effectively while using all of the appropriate PPE, but this is clearly a valuable skill that students need to master to work in most immunology laboratory settings. Having biohazard waste disposal containers (including sharps containers, if needed) available is also important. Be sure to check with the safety officer on your campus to be sure that you are in compliance with all applicable biohazard handling and disposal regulations.

Computer-based tools/simulations

In preparing for the lab, students learn about an epitope map that summarizes known, patient-derived CTL epitopes (see supplemental materials and https://www.hiv.lanl.gov/content/index). In the pre-lab preparation, it is useful to direct students to the algorithm site (http://www.cbs.dtu.dk/services/NetCTL/) and the epitope maps (http://www.hiv.lanl.gov/content/immunology/maps/maps.html). For context, KG also refers students to an HLA supertype summary because this configuration of the lab focuses on CD8 T cell epitopes. (http://www.hiv.lanl.gov/content/immunology/motif_scan/supertype2.html). However, this lab could be adapted to focus on CD4 T cell epitopes or antibody epitopes, with an appropriate selection of prediction algorithm. The genome map for HIV is also the source of HIV protein sequences that students can input into the epitope prediction algorithm (http://www.ncbi.nlm.nih.gov/nuccore/nc_001802.1). In the lab exercise, they compare the predictions of the algorithm against the map of known epitopes.

Even with the materials provided for students to review before lab, a pre-lab group review and discussion is helpful to be sure all students extracted the fundamental ideas they need to begin the analysis. Completing this lab requires students to work dynamically and collaboratively as unexpected situations arise in results (e.g., how should a predicted epitope that is offset by one or two amino acids from a mapped epitope be classified?). Sharing data at the end of the lab to produce a complete picture across all of the HIV proteins helps to emphasize the need for a consistent approach across groups, but does not necessarily require any one approach to produce a valid analysis. Students learn to identify and articulate the strengths and weaknesses of various approaches to categorizing and evaluating the data.

ELISA simulation lab (KG)

Because some of the technical complexity of the assay is initially masked by the use of a simulation kit, students can focus on the binary outcomes (positive or negative) and the implications of those outcomes, because the quantitative aspects of the assay are introduced in a later exercise. It also directs students to focus on conceptual considerations like the value of a confirmatory Western blot or the carry-over of maternal antibody from mother to infant, rather

than immediately focusing on evaluating a complex technique for technical errors. Some commercial ELISA kits developed for classroom use have scenario or simulation prompts included with the kit. The instructor can also develop scenarios around various outcomes. Depending on the clinical scenarios selected, this activity can spur discussion of a wide range of technical issues (including effective communication about safer sex practices, harm reduction models, vertical transmission of HIV, and persistence of maternal antibody in infants). An additional advantage of scenarios involving the last two points is the ability to illustrate the overlap between basic immunological principles and public health implications. One challenge in this area is that the instructor has responsibilities for both grading/evaluating student performance in the scenarios and leading the technical discussions to explore the scientific issues raised in each scenario to capitalize on student interest and engagement in the issues raised in a real-life context. This can easily spread an instructor too broadly to be effective, and delegation of one of the two areas of responsibility may be warranted.
