# Supplementary material for: Setting Up an Undergraduate Immunology Lab: Resources and Examples
Source: Front Immunol. 2019 Aug 27;10:2027. doi: 10.3389/fimmu.2019.02027 (PMC6718614; doi:10.3389/fimmu.2019.02027)
Supplement: Supplementary file 4 [file Data_Sheet_4.PDF]

Instructor notes for peritonitis lab

With heavy instructor guidance, students induce a sterile inflammatory response in the peritoneal cavities of C57BL/6 mice and then follow the immune response over time in both the blood and peritoneal cavity. In session 1, students learn how to handle mice and prepare and stain slides for blood differentials. Prior to the second lab session, the instructor injects 9 mice with 1 ml of 6% thioglycolate at various timepoints (3 mice each at 0, 24, and 48 hours). Students are taught how to inject the thioglycolate into 3 more mice at the beginning of the second session and differentials are taken from these mice 2 hours later during class. Additionally, after the instructor demonstrates the technique, students isolate cells from the peritoneal cavity of each mouse. These cells are counted using a hemocytometer and stained with fluorescently conjugated antibodies directed towards CD3, CD19, Gr1, and CD11b. The following lab session is devoted to acquiring the flow cytometry samples, analyzing the blood differentials, and pooling the class data. Students at Randolph-Macon College are taught the essentials of excel in the introductory biology course. Even so, it is usually necessary to spend at least one lab session helping students analyze, graph, and interpret the results.

After completing this lab, student should meet the learning objectives:

1. Identify lymphocytes, monocytes, and neutrophils in a blood smear,
2. Explain how and why blood differentials are taken, and
3. Describe the chronological order in which immune cells arrive at sites of inflammation.

This lab activity is typically done with 12 students working in 3 groups (4 students/group) in four 3-hour lab sessions. If the instructor prefers students to present their results, the beginning of the next lab session (session 5) should also be reserved this purpose. More students could be accommodated by increasing the size of groups or scaling up to incorporate a larger number of groups. Additionally, the number of lab sessions could be increased if more time is needed to teach students data analysis or if the instructor prefers to process a smaller number of mice per session.

A suggested timeline for each lab session and instructor prep is shown below.

Materials needed

Thioglycollate broth (Sigma, 70157)
Easy III Stain Kit (Azer Scientific, ES902-3)
Microscope slides
Microscope immersion oil
Lens paper
Ethanol-resistant marker
Anesthesia chamber and isoflurane (or another method for anesthesia)
Heat lamp
Razors
Ethanol wipes
Sharps disposal
Glass disposal

Compound microscopes with 100X Oil objectives
Gloves
Paper towels
C57BL/6 mice (at least 12 mice)
70% Ethanol squirt bottle
dissecting tools (one scissors, two tweezers)
10 ml syringes
23G needles
1X PBS
15 ml Falcon tubes
FACS Staining Solution
eppendorf tubes
CD3 – APC antibody
CD19 – PerCP-Cy5.5 antibody
Ly6C/G – FITC antibody
CD11b – PE antibody
p20, p200, p1000 tips
p20, p200, p1000 pipettes
4% paraformaldehyde
hemocytometer + cover slip
Trypan blue
hand tally
Flow cytometer

Instructor Setup

Three weeks (or more) prior to session 1:

- Order materials and at least 9 mice (to give the mice time to acclimate).
- Make 4% thioglycollate broth in ddH_2O, autoclave, and cool to room temperature. Wrap in aluminum foil to protect from light. As the solution ages, it will turn brown. Aged thioglycolate will elicit the most robust inflammatory response.
- Obtain sharps and glass disposal containers to dispose of razors and microscope slides after use.
- Set up anesthesia chamber and heat lamp station for obtaining blood samples from mice.

Two days prior to session 2:

- Inject 3 mice with 1 ml of sterile 4% thioglycollate intraperitoneal (i.p.).
- Dilute and aliquot antibodies and Fc-block. Aliquot other reagents needed for session 2.

One day prior to session 2:

- Inject 3 mice with 1 ml of sterile 4% thioglycollate i.p.

Day of session 2:

- Calibrate flow cytometer.

**Lab Session (3 hrs each)**	**In- lab Activities**
1	• Lab is introduced.<br>• Students learn how to handle mice and perform blood differentials on blood samples obtained from control (non-inflamed mice).
2	• Instructor can demonstrate and/or student volunteers inject 3 mice with sterile 4% thioglycollate broth (intra-peritoneal) – these mice will be the 2 hour time point.<br>• Obtain a blood smear from mice injected with TG previously. While slides are drying, begin harvesting cellular infiltrate (see below).<br>• Instructor euthanizes mice that were injected with TG one and two days prior to session and demonstrates how to harvest cellular infiltrate.<br>• Students harvest cellular infiltrate from 6 mice injected with TG previously and 3 mice injected with only saline. After two hours, the process is repeated for the mice injected during the session.<br>• Blood differentials are stained. During wait time, student will spin down the peritoneal cells harvested from all 12 mice, count the total number of cells, stain and fix them for next session.
3	• Students acquire their samples on the flow cytometer (group by group) and analyze their data. [The instructor draws gates with each group, but them applies the same gating strategy to all of the data and distributes it.]<br>• Students analyze blood differentials under the microscope, record their data, and compile it in excel.<br>• Students use excel to calculate averages, standard error of the means, perform one-way ANOVAs, and make graphs [MGB students are familiar with these techniques from R-MC's introductory biology course sequence.]<br>• Assign student groups discussion questions for presentations or papers.
4	• Usually one more lab session is required to help students analyze data, ask questions, and prepare for their presentation or paper.
5	• Reserve time at beginning of session for student presentations (if desired).

Example discussion questions to assign groups of students for presentations

- What types of junctions exist between endothelial cells lining blood vessel walls in the non-inflamed, resting condition? How do these junctions change during local inflammation?
- Relate your findings from the blood differentials with your findings from the flow cytometry data. Do the two sets of data “paint the same picture”? Why or why not?
- Map the route newly developed neutrophils must take to enter the inflamed peritoneal cavity. Begin in the bone marrow and end with the peritoneal cavity.

For more details on this model, please see: Liu, Z. (2011). Thioglycollate Induced Peritonitis. *Bio-101*: e84. DOI: 10.21769/BioProtoc.84.

For more information on collecting blood from mice, please see: Parasuraman, S, Raveendran, R, and Kesavan, R. J Pharmacol Pharmacother. 2010 Jul-Dec; 1(2): 87–93.

Instructor notes for phagocytosis lab

In this lab, broadly focused on communication between the innate and adaptive immune response, students analyze the effect of a particular substance on the ability of cultured macrophages to phagocytose latex beads.

The goals of the lab exercise are:
1. Compare and contrast monocytes and macrophages,
2. Evaluate flow cytometry data regarding the ability of cells to phagocytose fluorescently-tagged antigens
3. Design an experiment to test the impact of a particular substance on phagocytosis.

To accomplish these goals, students are first introduced to cell culture and are assigned a culture of THP-1 cells to take care of for the week. [Note: this lab could alternatively be conducted with primary bone-marrow derived macrophages isolated from mice.] Next, each group brainstorms a list of substances/compounds that they hypothesize might affect phagocytosis such as garlic extract, honey, particular vitamins/minerals, etc. The class agrees on a substance and designs an experiment to test the effect of the chosen substance on the ability of THP-1 cells to phagocytose FITC-coated latex beads. Students must consult the literature to determine methods of delivery, concentration, and the timing of application of the substance to the culture. Prior to the 2nd lab session, students set up 24-well plates with THP-1 cells + PMA (phorbol 12-myristate 13-acetate) so that during lab, students can incubate differentiated THP-1 cells (macrophages) with FITC-coated latex beads, process the cells for flow cytometry, and acquire their samples. [Note that if a flow cytometer is not available, an instructor may instead use a fluorescent microscope to assess phagocytosis.] The following lab session students compile the class data, analyze their results, and prepare their papers or presentations.

This lab activity is typically done with 12 students working in 3 groups (4 students/group) sharing the reagents provided in one Cayman Chemical Phagocytosis kit in two 3-hour lab sessions. If the instructor prefers students to present their results, the beginning of the next lab session should also be reserved this purpose. More students could be accommodated by increasing the size of groups or purchasing more kits to serve a larger number of groups. A suggested timeline for each lab session and instructor prep is shown below.

Materials needed

THP-1 cells (ATCC, TIB-202)
Phagocytosis kit (Cayman Chemical, 500290)
Cell stripper (VWR, 45000-668)
RPMI-1640 Medium (ATCC, 30-2001)
2-mercaptoethanol
Fetal Bovine Serum

T-75 Flasks
Trypan Blue
Transfer pipets
Marker
15 ml conical tubes
Eppendorf tubes
Serological pipets (5 ml, 10 ml, 25 ml)
p20, p200, p1000 tips
p20, p200, p1000 pipettes
24-well plates
Hemocytometer
70% ethanol
Phorbol 12-myristate 13-acetate (PMA) (VWR, 102515-692)
Compound microscope
Flow cytometer (or fluorescent microscope)
Bench top centrifuge
Tissue culture hood
37 degree/5% CO_2 incubator
37 degree water bath
Hand tally

THP-1 cell culture medium: 0.05 mM 2-mercaptoethanol and 10% fetal bovine serum in RPMI-1640 Medium (ATCC, 30-2001).

Instructor Setup

Four weeks (or more) prior to session 4:
- Order materials

Two weeks prior to session 4:
- Prepare THP-1 culture medium (see recipe above)
- Thaw THP-1 cells and begin culturing (you will need to increase the number of cells so that each student group can begin with at least 3 x 10^6 cells)

Three days after session 4
- Monitor student groups as they come in to "feed" their cells.

One day prior to session 5:
- Monitor student groups as they come in to set up their 24-well plates. They will likely need assistance.

Prior to sessions 4 and 5:
- Aliquot reagents

Prior to session 5:
- Calibrate flow cytometer.

Lab Session (3 hrs each)	In- lab Activities
**4**	• Lab is introduced.<br>• Students learn sterile technique and how to culture THP-1 cells.<br>• IMPORTANT: Class should discuss and decide on a reagent to treat THP-1 cells with PRIOR to this lab session. (Assign it during the previous lab activity so that the instructor has time to order the reagent and ready it for cell culture.) MGB has used Vitamin C, local honey, garlic extract, etc. in the past.
**3 days after session 4**	• Students feed THP-1 cells
**1 day before Session 5**	• Students set up 24-well plates for phagocytosis assay. They treat appropriate wells with PMA and with the agreed upon reagent (student choice).
**5**	• Students incubate appropriate wells with FITC-labeled latex beads and acquire cells on flow cytometer (instructors may instead opt to characterize phagocytosis using a fluorescent microscope.)
**6**	• Help students analyze data, ask questions, and prepare for their presentation or paper.
**7**	• Reserve time at beginning of next session for presentations (if desired)

Example discussion questions to assign groups of students for presentations

- What cell surface molecules are likely engaged when macrophages phagocytose IgG-FITC-latex beads? Be sure to include a diagram in your presentation.
- Describe the intracellular signaling pathway that PMA likely contributes to in THP-1 macrophages. Be sure to include a diagram.
- Describe the phenotypic changes that a cell goes through as it differentiates from a monocyte to a macrophage. Use diagrams/cartoons to support your answer.
- Suggest 3 additional treatments that we could apply to THP-1 cells to affect phagocytosis (either enhance or inhibit). Be sure to use peer-reviewed papers to back-up your suggestion. Include a table with the treatment and the likely effect.
